# Monitoring oyster culture rafts and seagrass meadows in Nagatsura-ura Lagoon, Sanriku Coast, Japan before and after the 2011 tsunami by remote sensing: their recoveries implying the sustainable development of coastal waters

**DOI:** 10.7717/peerj.10727

**Published:** 2021-01-14

**Authors:** Hiroki Murata, Motoyuki Hara, Chinatsu Yonezawa, Teruhisa Komatsu

**Affiliations:** 1 Graduate School of Agricultural Science, Tohoku University, Sendai, Japan; 2 Port and Harbor Bureau, City of Yokohama, Yokohama, Japan; 3 Tohoku Ecosystem-Associated Marine Science, Graduate School of Agricultural Science, Tohoku University, Sendai, Japan; 4 Faculty of Commerce, Yokohama College of Commerce, Yokohama, Japan; 5 Atmosphere and Ocean Research Institute, The University of Tokyo, Kashiwa, Japan

**Keywords:** Remote sensing, Blue infrastructure, Aquaculture facility, Seagrass meadow, Sustainability, Civil work design, High-resolution satellite image, Unmanned aerial vehicles

## Abstract

**Background:**

Coastal ecosystems are blue infrastructures that support coastal resources and also aquaculture. Seagrass meadows, one of coastal ecosystems, provide substrates for epiphytic diatoms, which are food resources for cultured filter feeder organisms. Highly intensive coastal aquaculture degrades coastal environments to decrease seagrass meadows. Therefore, efficient aquaculture management and conservation of seagrass meadows are necessary for the sustainable development of coastal waters. In ria-type bays, non-feeding aquaculture of filter feeders such as oysters, scallops, and ascidians are actively practiced along the Sanriku Coast, Japan. Before the 2011 Great East Japan Earthquake, the over-deployment of oyster culture facilities polluted the bottom environment and formed an hypoxic bottom water layer due to the organic excrements from cultured oysters. The tsunami in 2011 devastated the aquaculture facilities and seagrass meadows along the Sanriku Coast. We mapped the oyster culture rafts and seagrass meadows in Nagatsura-ura Lagoon, Sanriku Coast before and after the tsunami and monitored those and environments after the tsunami by field surveys.

**Methods:**

We conducted field surveys and monitored the environmental parameters in Nagatsura-ura Lagoon every month since 2014. We used high-resolution satellite remote sensing images to map oyster culture rafts and seagrass meadows at irregular time intervals from 2006 to 2019 in order to assess their distribution. In 2019, we also used an unmanned aerial vehicle to analyze the spatial variability of the position and the number of ropes suspending oyster clumps beneath the rafts.

**Results:**

In 2013, the number and distribution of the oyster culture rafts had been completely restored to the pre-tsunami conditions. The mean area of culture raft increased after the tsunami, and ropes suspending oyster clumps attached to a raft in wider space. Experienced local fishermen also developed a method to attach less ropes to a raft, which was applied to half of the oyster culture rafts to improve oyster growth. The area of seagrass meadows has been expanding since 2013. Although the lagoon had experienced frequent oyster mass mortality events in summer before the tsunami, these events have not occurred since 2011. The 2011 earthquake and tsunami deepened the sill depth and widened the entrance to enhance water exchange and improve water quality in the lagoon. These changes brought the expansion of seagrass meadows and reduction of mass mortality events to allow sustainable oyster culture in the lagoon. Mapping and monitoring of seagrass meadows and aquaculture facilities via satellite remote sensing can provide clear visualization of their temporal changes. This can in turn facilitate effective aquaculture management and conservation of coastal ecosystems, which are crucial for the sustainable development of coastal waters.

## Introduction

As the global population continues to increase at a rapid rate, the food produced by aquaculture is partially expected to compensate for the growing food demand (Naylor et al., 2000; World Bank, 2013; United Nations, Department of Economic and Social Affairs, Population Division, 2019). Coastal aquaculture is carried out in coastal waters and is supported by coastal ecosystems. Seagrass meadows, one type of coastal ecosystems, provide substrates for epiphytic diatoms, which are food resources for filter feeder species cultured in estuaries and lagoons, e.g., clams *Ruditapes philippinarum* (Adams & Reeve, 1848-1850) and oysters *Magallana gigas* (Thunberg, 1793) (Kasim & Mukai, 2006; Komorita et al., 2014; Lebreton et al., 2011). A recent study found that oyster spats cultured on tidal flats near seagrass meadows had higher nitrogen contents and higher *δ*^13^C ratios than spats cultured with a raft in an offshore water (Hori et al., 2018). This suggested that the coastal ecosystems enable oysters to use both pelagic and benthic pathways of primary production. Moreover, seagrass meadows perform ecological roles such as spawning substrates, nurseries area for juvenile organisms, a feeding area for various living organisms, and a refuge from predation (Heck, Hays & Orth, 2003; Horinouchi, 2007; Kharlamenko et al., 2001). They also help to mitigate climate change through the capture and storage of organic carbon known as blue carbon (Nellemann et al., 2009). Coastal ecosystems are also referred to as blue infrastructures because their habitats and networks can be managed like those of green infrastructures (i.e., terrestrial ecosystems) (Komatsu et al., 2017; Komatsu et al., 2019). However, over the years, coastal aquaculture development has degraded some coastal ecosystems (Delgado et al., 1999; Huitric, Folke & Kautsky, 2002; Ilman et al., 2016). Therefore, the conservation of coastal ecosystems as well as the efficient management of aquaculture is crucial for the sustainable development of coastal waters (Komatsu et al., 2012).

In Japan, aquaculture is carried out in the areas designated by the prefectural governors according to the Demarcated Fishery Rights (the rights to operate a demarcated fishery area under the Fishery Act of Japan). These areas are directly managed by a local Fishery Cooperative Association (FCA), which is organized by local fishermen in front of the areas on behalf of a governor. A prefectural government decides on aquaculture areas (as map polygons), while local FCAs decide on the target species and the number of aquaculture facilities. Aquaculture is therefore managed by the FCAs following a “bottom-up” structure in Japan (Komatsu & Aoki, 2020). Nevertheless, in general, FCAs do not know the exact locations of aquaculture facilities, as it is difficult to gather information about locations of aquaculture facilities solely from field surveys when they are numerous (Komatsu et al., 2012; Murata, Komatsu & Yonezawa, 2019). Since the sustainable use of coastal waters is necessary for Sustainable Development Goal 14 saying “Conserve and sustainably use the ocean, sea and marine resources for sustainable development” accurately mapping aquaculture facilities by type in a demarcated fishery right area contributes to SDG14 through their management.

**Figure 1:**
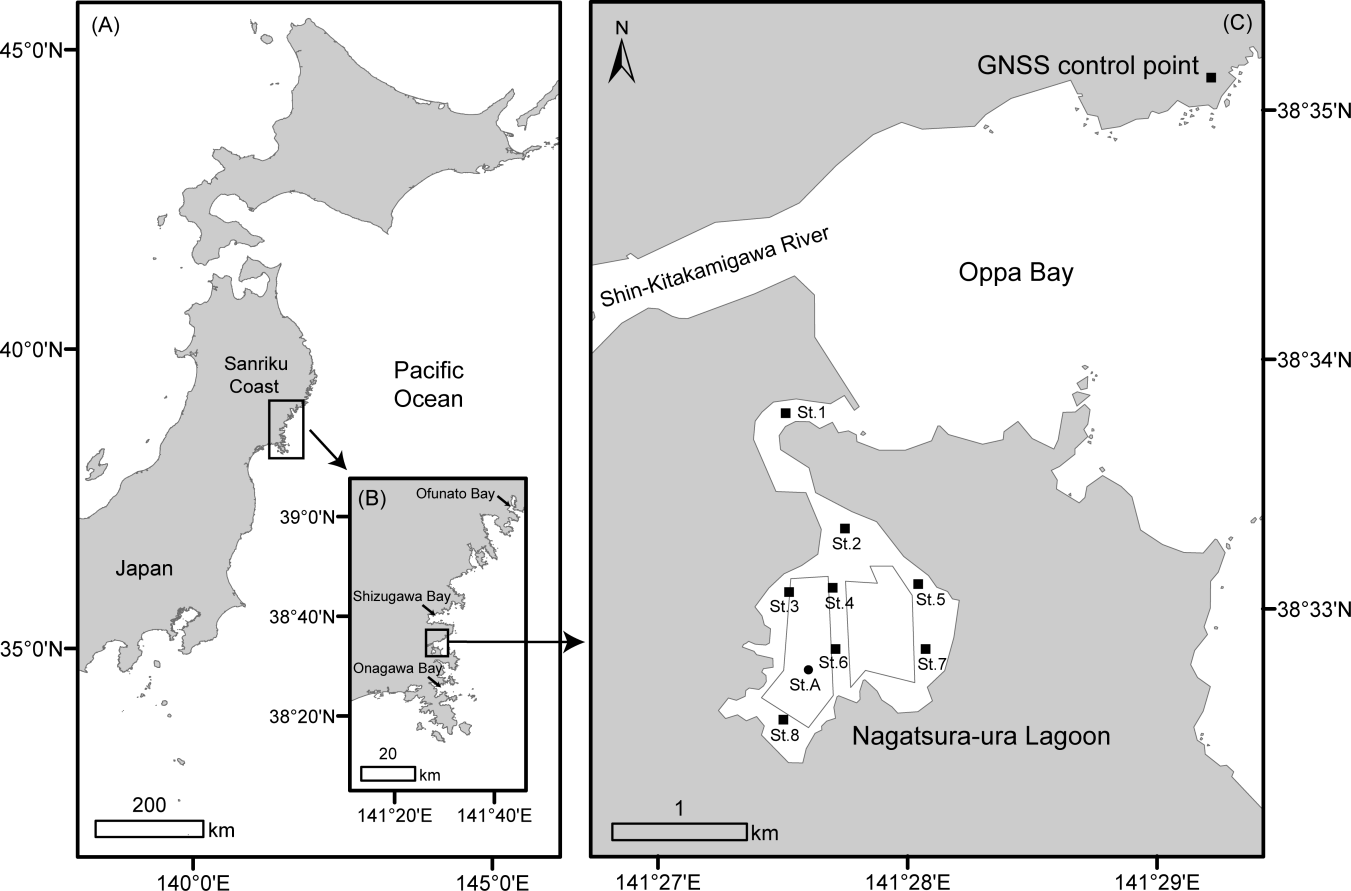
Map of the study site. Map showing location of Nagatsura-ura Lagoon, Sanriku Coast in northeast Honshu Island, Japan (A–B), and field survey stations (St.1 to St.8 and St. A), the two demarcated fishery right areas and the GNSS control point (C).

As shown in Fig. 1, ria-type bays are distributed along the Sanriku Coast in the northeastern Honshu Island facing the Pacific Ocean. Non-feeding aquaculture of filter feeders, such as oysters, scallops *Mizuhopecten yessoensis* (Jay, 1857), ascidians *Halocynthia roretzi* (Drasche, 1884), and seaweed like *Undaria pinnatifida* (Harvey) Suringar (1873) and *Saccharina japonica* (Areschoug) Lane et al. (2006), are typically conducted on ria-type bays on the Sanriku Coast because of their calm oceanographic conditions and large water exchange. Non-feeding aquaculture does not typically influence the coastal environment, whereas feeding aquaculture causes eutrophication and bottom water deterioration (Yokoyama, 2003; Pergent-Martini et al., 2006). However, aquaculture can pollute the coastal environment when the biodeoposition caused by cultured organisms exceeds their removal through decomposition and by water exchange. In Shizugawa Bay, which is located in the south of the middle part of Sanriku Coast, highly dense distributions of oyster culture longlines polluted the bottom environment when the high organic loads from increased oyster excrement produced an hypoxic water layer because of bacterial oxygen consumption (Nomura et al., 1996). This subsequently prevented oyster growth (Komatsu et al., 2018a). In Ofunato Bay, the middle part of Sanriku Coast, the organic matter in oyster excrement from culture rafts caused an hypoxic water layer near the bottom in summer as seen in Shizugawa Bay, and resulted in decreased oyster productivity (Miyazawa & Hayakawa, 1994). Several researchers pointed out that the construction of breakwaters at the entrance decreased the water circulation and increased the stratification by preventing vertical mixing of seawater in the bay (Hayakawa, Kobayashi & Izawa, 2001; Kakehi et al., 2017).

The Great East Japan Earthquake on March 11, 2011, triggered the 2011 tsunami on the Sanriku Coast that brought significant damage to fishery infrastructure, including fishing boats, fishing ports, and aquaculture facilities. The Ministry of Agriculture, Forestry Fisheries (2020) reported a virtually complete infrastructure recovery nine years after the disaster. The utilization of demarcated fishery right areas has changed after the tsunami in some ria-type bays such as Shizugawa Bay (Komatsu & Aoki, 2020) and Onagawa Bay (Fujii et al., 2019) to reduce aquaculture facilities. Coastal ecosystems also suffered significant damage on tidal flats (Suzuki, 2012) and seagrass meadows except seaweed beds (Komatsu, 2012; Komatsu et al., 2015; Sakamoto et al., 2012; Sasa et al., 2012). The Biodiversity Center of Japan (2015) reported that the area of seagrass along the Sanriku Coast before the tsunami had decreased during the 2012–2014 monitoring period. Consequently, mapping seagrass meadows is a very practical method to monitor the recovery process in coastal ecosystems after tsunamis, although only a few studies have attempted this monitoring (e.g., Komatsu et al., 2018b; Tsujimoto et al., 2016). Since bottom depths were changed by co-seismic land subsidence and post-seismic uplift after the tsunami (Geospatial Information Authority of Japan, 2020), the lower depth limits of seagrass meadows limited by light availability enough for survivals of seagrasses (Duarte, 1991; Komatsu & Tatsukawa, 1998; Sakanishi & Komatsu, 2017) are also influenced by a height of water column above the bottom depending on the bottom depth. Coastal reconstructions, including the construction of large seawalls, breakwaters, and roads, could also affect the shallow bottom near the coast where ecotone between land and the sea is extended in coastal waters (Komatsu et al., 2012; Komatsu et al., 2018a). Therefore, long-term monitoring of post-tsunami coastal ecosystems were essential to assess the coastal environments.

Satellite remote sensing can be used to map broad areas at once. In particular, seagrass meadows have been successfully mapped using high-resolution satellite images. For example, Roelfsema et al. (2014) used a semi-automated object-based image analysis on WorldView-2, IKONOS, and Quickbird-2 multispectral satellite images at 2.4–4.0 m resolutions to map seagrass cover, species, and biomass in the Eastern Banks of Moreton Bay, Australia. Tsujimoto et al. (2016) used GeoEye-1 and WorldView-2 multispectral satellite images at 2.0 m resolution to map seagrass meadows before and after the 2011 tsunami in Matsushima Bay on the Sanriku Coast. Aquaculture facilities have also been mapped via high-resolution satellite remote sensing. For example, Komatsu et al. (2002) used IKONOS multispectral images at 4.0 m resolution and panchromatic images at 1.0 m resolution to detect raft and longline aquaculture facilities in Yamada Bay on the Sanriku Coast. Moreover, Komatsu et al. (2012) later applied ALOS multispectral images at 10.0 m resolution and panchromatic images at 2.5 m resolution for the analysis of aquaculture facilities in the same region. Fu et al. (2019) used WorldView-2 multispectral satellite images at 2.0 m resolution and panchromatic images at 0.5 m resolution to map cage culture and raft culture areas around Sandu Island in Fujian Province, China. Recently, it was reported that unmanned aerial vehicles (UAV) were an efficient tool to observe coastal waters because the images they captured at any time of the day had better resolution than satellite images.

In this study, we aimed to reveal the recovery and changes of coastal environments and aquaculture conditions from the 2011 tsunami through the long-term monitoring. In addition, we aimed to confirm whether remote sensing is an effective method to manage coastal waters for sustainable development. We monitored the environmental parameters of demarcated fishery right area by field surveys after the 2011 tsunami and map oyster culture rafts and seagrass meadows in a lagoon in Sanriku Coast with use of archived satellite images for retrospective analysis. Furthermore, we conducted UAV observation to count and measure the number and position of ropes attached to the rows of oyster culture rafts to know a method for culturing oysters in the lagoon after the tsunami. Finally, we discuss spatio-temporal changes of oyster culture rafts and seagrass meadows there before and after the tsunami.

## Materials & Methods

### Study site and field survey

**Figure 2:**
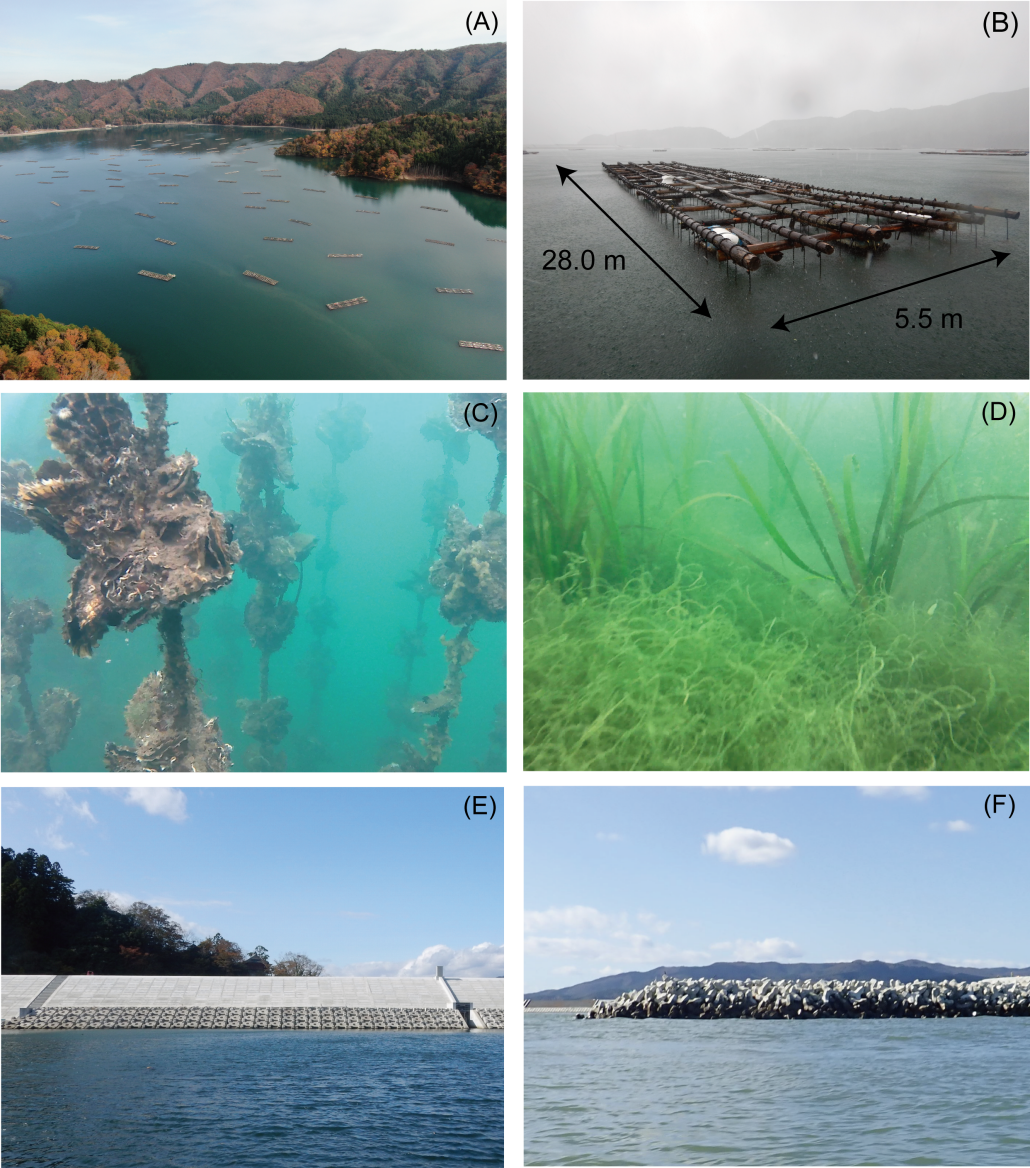
Photographs of the oyster culture rafts, seagrass, seawall, and breakwaters from Nagatsura-ura Lagoon, Sanriku Coast, Japan. Distribution of oyster culture rafts (A), a close-up of a raft (B), ropes suspending clumps attached to a oyster culture raft (C), *Zostera marina* with green seaweed *Cladophora* sp. (D), seawall along the coast of the lagoon (E), and breakwaters at the entrance of the lagoon (F) in Nagatsura-ura Lagoon, Sanriku Coast. Photographs by (A–E) Hiroki Murata, and (F) Motoyuki Hara.

We selected Nagatsura-ura Lagoon as a study site which has an area of 1.41 km^2^ and is located near the Shin-Kitakamigawa River and Oppa Bay in the southern Sanriku Coast (Fig. 1) because of its widespread distribution of oyster culture rafts, one of the most important oyster productions in the bays of Miyagi Prefecture and seagrass meadows (Fig. 2A). Miyagi Prefecture registered a total of 57.9 ha of two demarcated fishery right areas for aquaculture (Fig. 1) which managed by the Kahokucho Branch of the Miyagi FCA. According to the FCA branch, the standard size of an oyster culture raft is 28.0 × 5.5 m (Fig. 2B). A single raft comprised 15 buoys connected to 6 rows and 14 columns of cedar logs and is generally used for 5 to 10 years.

For oyster cultures, oyster clumps consisting of scallop shells to which oyster larvae attached (called oyster seed collectors) are suspended on ropes tied to rafts (Fig. 2B) and then submerged up to the maximum depth of approximately 5 m (Fig. 2C). We had a verbal agreement to research the oyster culture rafts and the production data of oyster were also provided from Mr. Kazushi Abe, who is a manager of Kahokucho Branch of the Miyagi FCA. In the Sanriku Coast, oysters grow to commercial selling sizes within 2–3 years. In contrast, oysters in Nagatsura-ura Lagoon reach commercial sizes in less than a year before they are harvested. The high productivity in Nagatsura-ura Lagoon is maintained by longer algal bloom periods than that of other ria-type bays in Sanriku Coast. The algal blooms feed cultured oysters, and the blooming period typically occurs from March to September in Nagatsura-ura Lagoon (Kaneko, Okumura & Hara, 2019). The annual schedule of oyster aquaculture in Nagatsura-ura Lagoon occurs as follows: (1) seed oysters attached to scallop shells are purchased from other bays in Miyagi Prefecture from January to February; (2) clumps of seed oysters attached to scallop shells are deployed to rafts already fixed in a demarcated fishery right area in Nagatsura-ura Lagoon from February to March; (3) attached organisms are removed from oyster clumps and ropes during the culture in the lagoon, especially in July when organisms attached to clumps and ropes increase; and (4) fully-grown oysters are harvested and sold from November to the following April.

The 2011 earthquake caused large-scale co-seismic crustal deformation in the Sanriku coastal region. The GNSS Earth Observation Network (GEONET) by the Geospatial Information Authority of Japan (GSI) recorded land deformation along the Sanriku Coast including Nagatsura-ura Lagoon (Fig. 1). Areas along the Sanriku Coast had large-scale subsidence and then have since undergone long-term uplifting (Imakiire & Koarai, 2012). The relative land deformation occurred at a rate of 4–8 cm per year, resulting in a total uplift of 43 cm between February 2012 and February 2020. Moreover, ground-level is still 33 cm lower in February 2020 than in February 2011.

We have conducted field surveys at a fixed station (Fig. 1, St. A) every month since 2014 and monitored environmental parameters—temperature, salinity, dissolved oxygen, and chlorophyll *a*—using a RINKO-Profiler (JFE Advantech Co., Ltd). Since 2013, the same surveys at 8 stations (Fig. 1 St. 1–8) have been added to the previous station once or twice a year in summer and/or winter, and the distribution of seagrass meadows was visually monitored from the boat with a portable GPS (Table 1). We also conducted a field survey using UAV on November 20–22, 2019, just before oyster harvesting. The distribution of oyster culture rafts and the positions of ropes suspending clumps attached to a raft were recorded. The seagrass meadows were also observed using a video camera lowered from the boat. In addition, in order to check the difference in local water quality, we used a RINKO-Profiler to observe chlorophyll *a* inside and outside a raft from August 1 to October 28, 2019.

**Table 1:** Grand truth data of seagrass meadows from 8 field survey stations shown in Fig. 1 in Nagatsura-ura Lagoon.

Date	St.1	St.2	St.3	St.4	St.5	St.6	St.7	St.8
September 26, 2013	×	×	×	×	×	×	×	×
February 22, 2014	×	×	×	×	×	×	×	×
July 7, 2014	×	×	×	×	⊚	×	∘	⊚
July 7, 2015	×	×	⊚	×	⊚	×	×	×
July 21, 2016	×	∘	∘	∘	⊚	×	×	×
March 7, 2017	×	∘	×	×	∘	×	×	×
September 8, 2017	×	∘	×	×	∘	×	×	×
March 13, 2018	×	∘	∘	△	×	△	∘	×
September 21, 2018	×	⊚	⊚	×	⊚	×	⊚	×
February 18, 2019	×	⊚	⊚	×	⊚	×	⊚	×

**Notes.**

× Not confirmed.

⊚ Strongly confirmed.

∘ Confirmed.

△ Floating seagrass confirmed.

### High-resolution satellite remote sensing and UAV images

We obtained high-resolution satellite images of multispectral bands at spatial resolutions of 1.2–2.4 m and panchromatic bands at resolutions of 0.3–0.8 m of QuickBird-2, GeoEye-1, and WorldView-2,3 from 2006 to 2019 for mapping seagrass meadows and oyster culture rafts, respectively (Table 2). One Google Earth image in Nagatsura-ura Lagoon on April 6, 2011 (Google) was also used to map seagrass meadows and oyster culture rafts just after the 2011 tsunami. In addition, we detected the coastline changes using satellite images. A UAV Mavic2 Zoom (DJI Co., Ltd) was used to collect RGB images of oyster culture rafts on November 20–22, 2019 with a flight altitude of approximately 25 m with a spatial resolution of 2 cm by selecting oyster culture rafts randomly across Nagatsura-ura Lagoon.

**Table 2:** Details of the high-resolution satellite images used in this study.

Imagery date	Satellite	Resolution (m)	
		Multispectral band	Panchromatic band
November 9, 2006	QuickBird-2	2.4	0.8
May 8, 2007	QuickBird-2	2.4	0.8
June 25, 2010	GeoEye-1	2.0	0.5
April 6, 2011	Google Earth	–	–
February 22, 2012	GeoEye-1	2.0	0.5
April 14, 2013	GeoEye-1	1.6	0.4
April 19, 2016	WorldView-3	1.2	0.3
March 19, 2017	WorldView-2	2.0	0.5
June 7, 2018	WorldView-3	1.2	0.4
May 8, 2019	WorldView-2	2.0	0.5

### Data analysis

**Figure 3:**
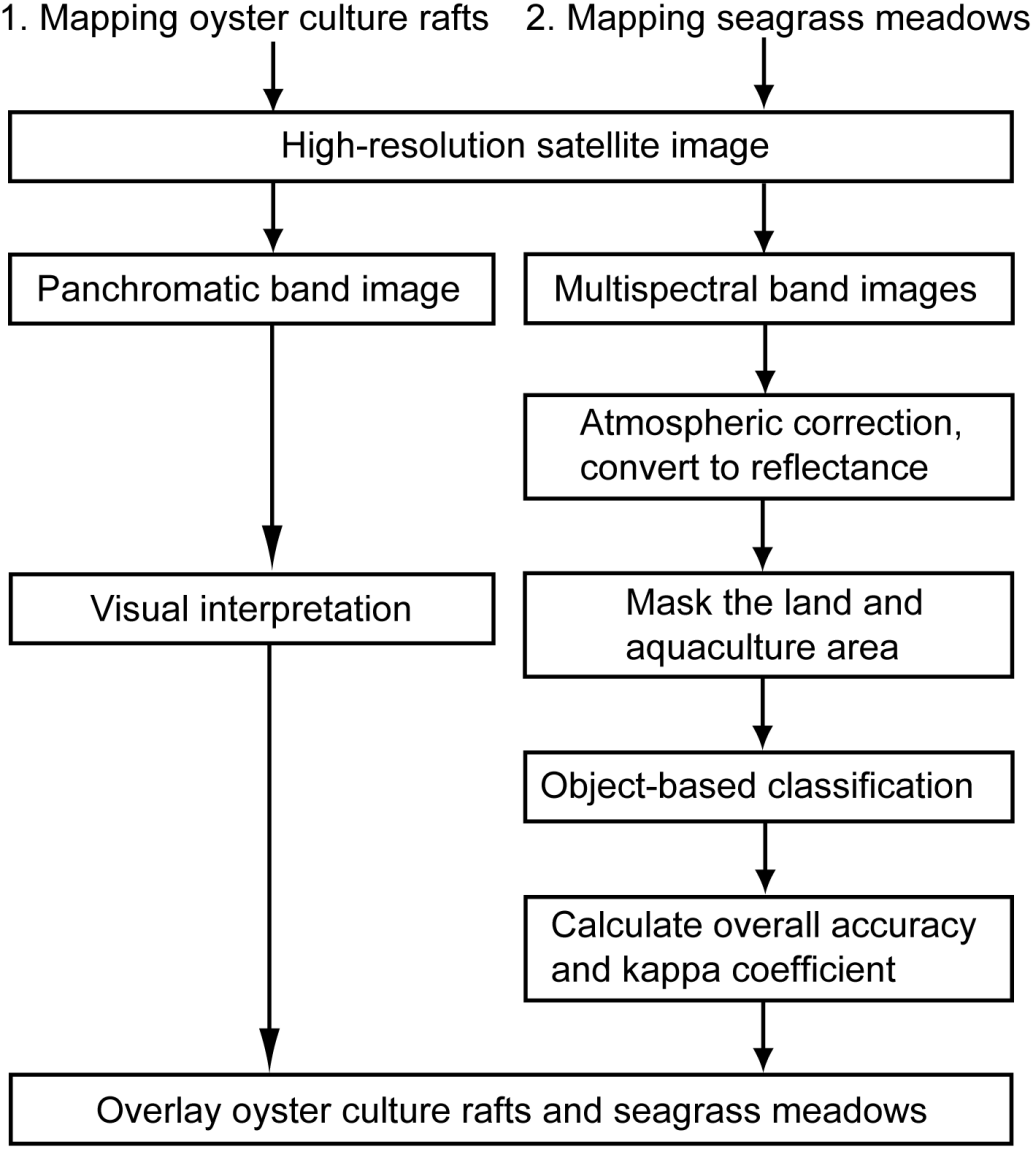
Flowchart of the analysis procedure for the mapping of oyster culture rafts and seagrass meadows using high-resolution satellite images.

The flowchart of the analysis procedure is shown in Fig. 3. We used ENVI 5.5 (Harris Geospatial) and ArcGIS 10.5 (ESRI) software for image processing and analysis. Oyster culture rafts were mapped by visual interpretation based on the panchromatic band images (left column of Fig. 3). The number of oyster culture rafts and their area were also obtained from distributions and areas of oyster culture rafts mapped from the images. The mean area of the rafts per image was calculated based on the total area of oyster culture rafts and the number of rafts mapped. Rafts that had detached from anchors in the 2011 tsunami and that remained on the sea surface and/or entangled with floating debris just after the 2011 tsunami, they were excluded from the mapping. Seagrass meadows were extracted from the RGB and near-infrared bands by the following procedure (right column of Fig. 3): (1) digital numbers were converted to surface reflectance values by atmospheric correction; (2) land and aquaculture areas were masked to prevent incorrect analysis; (3) supervised /object-based classifications were applied to surface reflectance images to classify pixel objects into three classes: shallow sand, seagrass meadows, and seawater areas; and (4) the overall accuracy and kappa coefficient were calculated to evaluate the classification accuracy. The overall accuracy was calculated by summing the number of correctly classified values and then dividing that sum by the total number of values. The kappa coefficient measures the agreement between the classification and the grand truth data. A kappa value of 1 represents perfect agreement, while a value of 0 represents no agreement (Kraemer, 2014). Supervised classification requires the use of ground truthed data obtained from field surveys. As the yearly field survey for this study began in 2013, training and evaluation data of seagrass, seawater, and sand were extracted by the visual interpretation of RGB composite images before 2013. RGB images obtained by UAV were analyzed by visual interpretation to localize the positions of ropes on a raft and count the number of the ropes suspending oyster clumps.

## Results

### Environmental parameters

**Figure 4:**
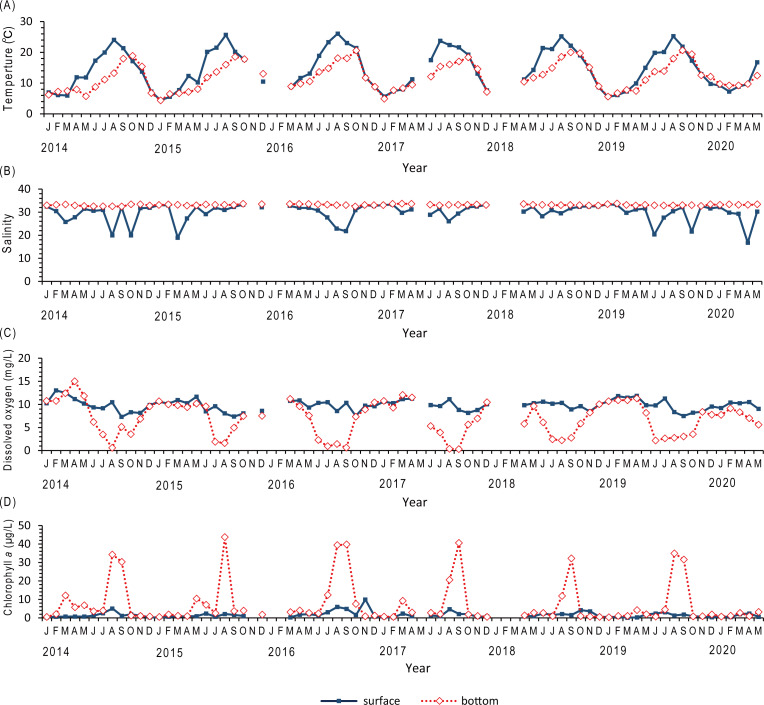
Environmental parameters from the field surveys in Nagatsura-ura Lagoon. Time series of water temperature (A), salinity (B), dissolved oxygen (C), and chlorophyll *a* (D) monthly measured at the sea surface and bottom layers of Nagatsura-ura Lagoon by the field surveys from January 2014 to May 2020.

The environmental parameters inside the demarcated fishery right area were observed (Fig. 1, St.A). Water temperatures in the surface and bottom layers varied from 4.4 to 26.0 °C and from 4.4 to 20.7 °C, respectively. That in the surface layer were higher than those in the bottom layer between March and September (Fig. 4A). Salinities in the surface and bottom layer fluctuated from 16.7 to 33.4 and from 32.4 to 33.7, respectively. That in the bottom layer was stable throughout a year, whereas that in the surface layer was lower than in bottom layer for several months generally in spring due to snow melting season and summer during rainy and typhoon seasons in a year (Fig. 4B). Dissolved oxygen contents in the surface and bottom layers varied from 7.3 to 13.0 mg L^−1^ and from 0.3 to 15.0 mg L^−1^, respectively. The dissolved oxygen content was significantly low in summer, and hypoxia water was found in the bottom layer from June to October (Fig. 4C). Chlorophyll *a* contents in the surface and bottom layers changed from 0.1 to 10.0 µg L^−1^ and from 0.37 to 43.75 µg L^−1^, respectively. The chlorophyll *a* content in the bottom layer was significantly higher from July to September (Fig. 4D).

**Figure 5:**
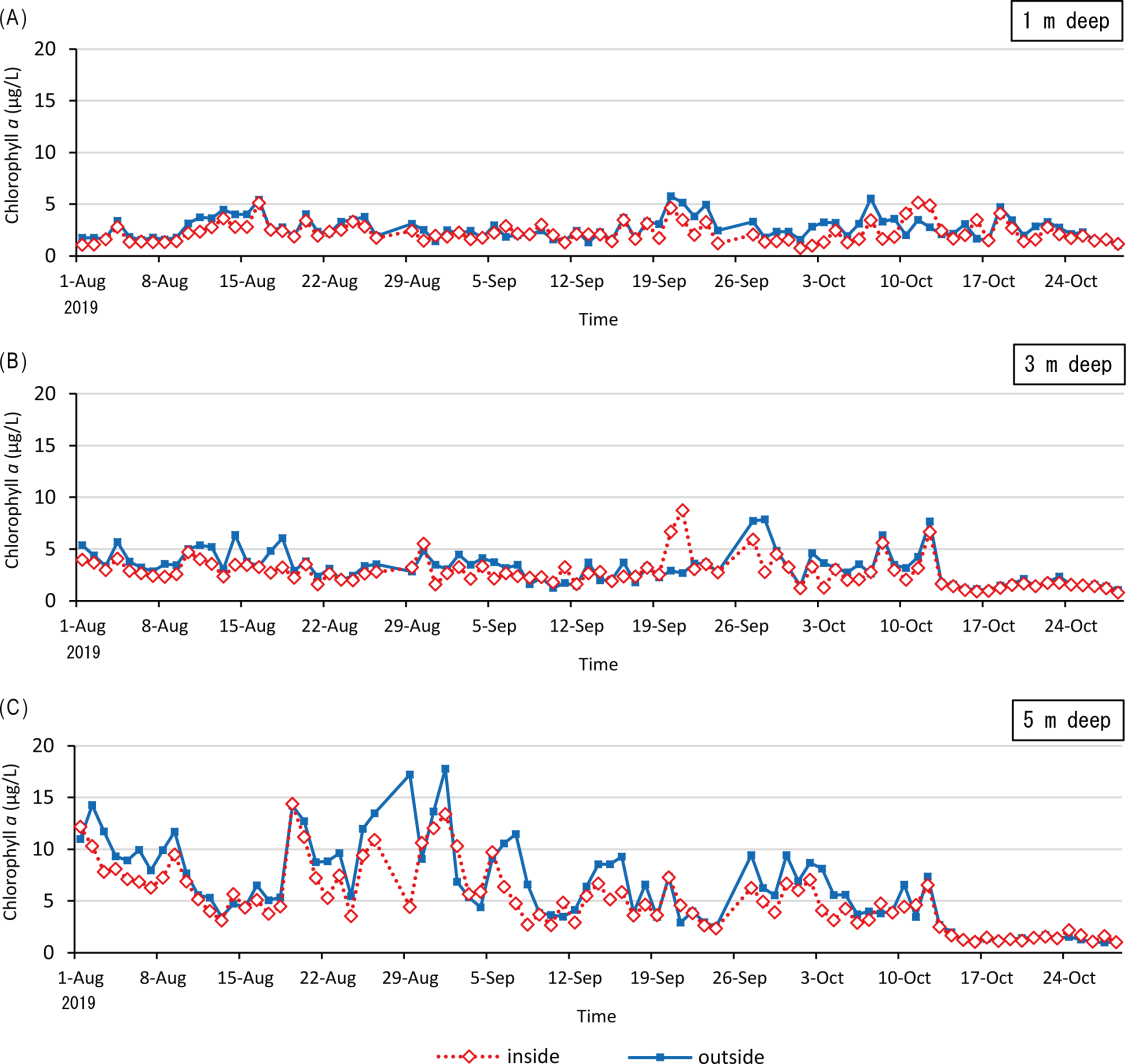
The monitoring of chlorophyll *a* contents inside and outside the oyster culture raft. Time-series of chlorophyll *a* contents inside (red dashed line with diamonds) and outside (blue solid line with squares) a raft in layers of 1 m deep (A), 3 m deep (B), and 5 m deep (C).

A difference in the chlorophyll *a* content was found between inside and outside the raft (Fig. 5) of which the ropes were attached to 6 rows. The mean chlorophyll *a* contents at the depths of 1, 3, and 5 m were 2.26 (SD = 0.95), 2.86 (SD = 1.36), and 5.36 µg L^−1^ (SD = 3.15) inside the raft, respectively; and at those of 2.73 (SD = 1.08), 3.39 (SD = 1.59), and 6.44 µg L^−1^ (SD = 3.92) outside the raft, respectively. At all the depths observed, the mean chlorophyll *a* content inside the raft was lower than that outside the raft. In addition, at low depths, the chlorophyll *a* contents were high both inside and outside the raft.

**Figure 6:**
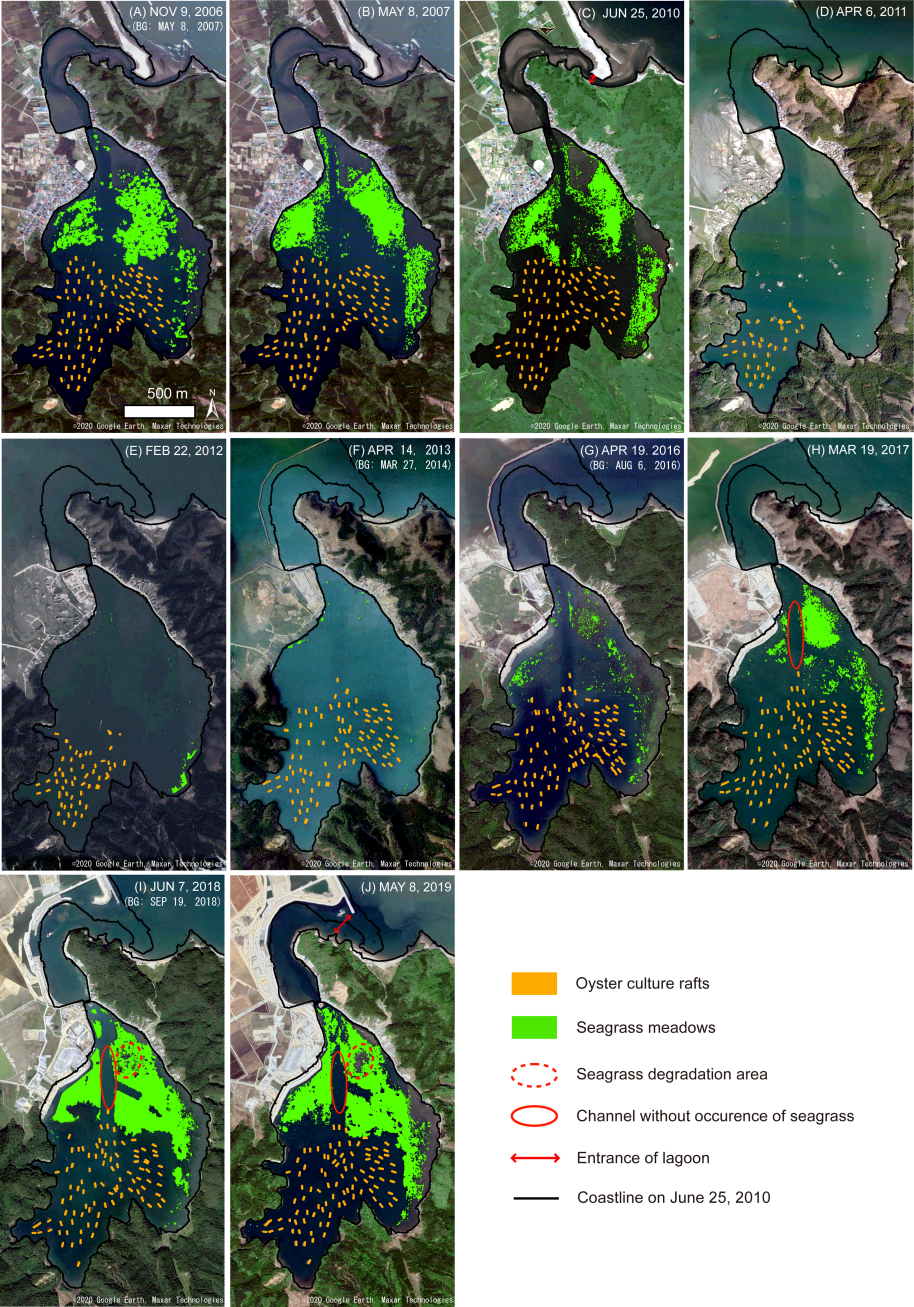
Mapping results of the oyster culture rafts and seagrass meadows, and the changes in coastline morphology in Nagatsura-ura Lagoon. Thematic maps showing oyster culture rafts (orange rectangles) and seagrass meadows (green areas) extracted by analyzing satellite images taken on November 9, 2006 (A), May 8, 2007 (B), June 25, 2010 (C), April 6, 2011 (D), February 22, 2012 (E), April 14, 2013 (F), April 19, 2016 (G), March 19, 2017 (H), June 7, 2018 (I) and May 8, 2019 (J). The red dashed ellipses, the red solid ellipses, red double-sided arrow and black lines are seagrass meadows degradated after 2017, the channel without the occurrence of seagrass, the entrance of the lagoon and the coastline on June 25, 2010, respectively. The background images (BG) showing the closest date available on Google Earth, copyrights belong to 2020 Google Earth, Maxar Technologies.

### Spatio-temporal changes in oyster culture rafts and oyster production

The numbers of 107, 103, and 98 oyster culture rafts were distributed in the Nagatsura-ura Lagoon on November 9, 2006, May 8, 2007, and June 25, 2010, respectively (Figs. 6A–6C, Table 3). The numbers of 40 and 63 oyster culture rafts were observed on April 6, 2011 just after the tsunami (Fig. 6D, Table 3) and on February 22, 2012, one year after the tsunami (Fig. 6E, Table 3), respectively and corresponded to 41% and 64% to the total number of the oyster culture rafts on June 25, 2010, respectively. Oyster culture rafts were increased to 96 rafts two years after the tsunami on April 14, 2013 (Fig. 6F, Table 3). The total number of the rafts was varied between 93 and 102 from April 14, 2013 to May 8, 2019 (Figs. 6F–6J, Table 3), and remained relatively constant after 2013.

**Table 3:** Mapping results of the oyster culture rafts and seagrass meadows in Nagatsura-ura Lagoon. The total number and mean area of a raft observed by visual interpretation, and seagrass area, overall accuracy, and kappa coefficient of seagrass classification obtained by supervised/object-based image classification.

Imagery date	Oyster culture rafts			Seagrass meadows		
	Number	Mean size (m^2^)	Standard deviation (m^2^)	Area (ha)	Overall accuracy (%)	Kappa coefficient
November 9, 2006	107	144.2	22.4	22.6	*	*
May 8, 2007	103	140.7	18.4	27.0	*	*
June 25, 2010	98	135.1	21.3	24.5	*	*
April 6, 2011	40	121.1	14.1	0.0^**^	–	–
February 22, 2012	63	107.7	26.2	0.8	*	*
April 14, 2013	96	142.8	26.4	0.3	*	*
April 19, 2016	102	148.0	22.9	4.1	71.7	0.57
March 19, 2017	100	151.5	25.6	12.5	85.0	0.75
June 7, 2018	93	151.0	18.1	33.5	84.4	0.74
May 8, 2019	97	165.2	28.6	36.1	83.9	0.74

**Notes.**

* The overall accuracy and kappa coefficient could not be evaluated due to the absence of ground truth data.

** Seagrass were not observed.

The mean areas of oyster culture rafts were 135.1 m^2^(SD = 21.3), 151.0 m^2^ (SD = 18.1), and 165.2 m^2^(SD = 28.6) on June 25, 2010, June 7, 2018, and May 8, 2019, respectively and increased from 2012 to 2019. Since the mean raft area was increased after the tsunami and the total number of oyster culture rafts on June 25, 2010 was almost equal to those of April 14, 2013 to May 8, 2019, therefore the total raft area was increased from 2010 to 2019.

**Figure 7:**
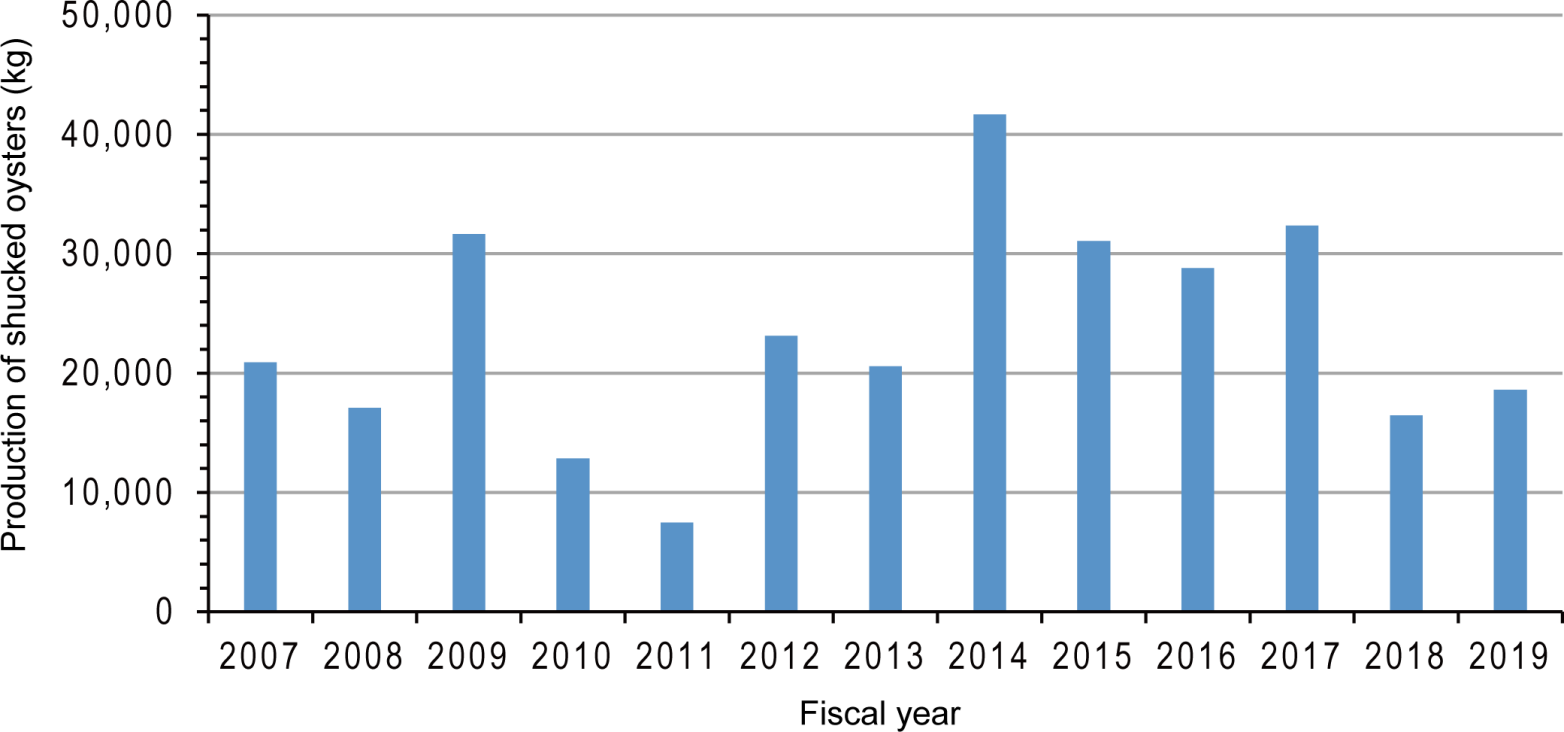
Production of shucked oysters from 2007 to 2019 in Nagatsura-ura Lagoon, Sanriku Coast, Japan (provided by Kahokucho Branch of the Miyagi FCA).

**Figure 8:**
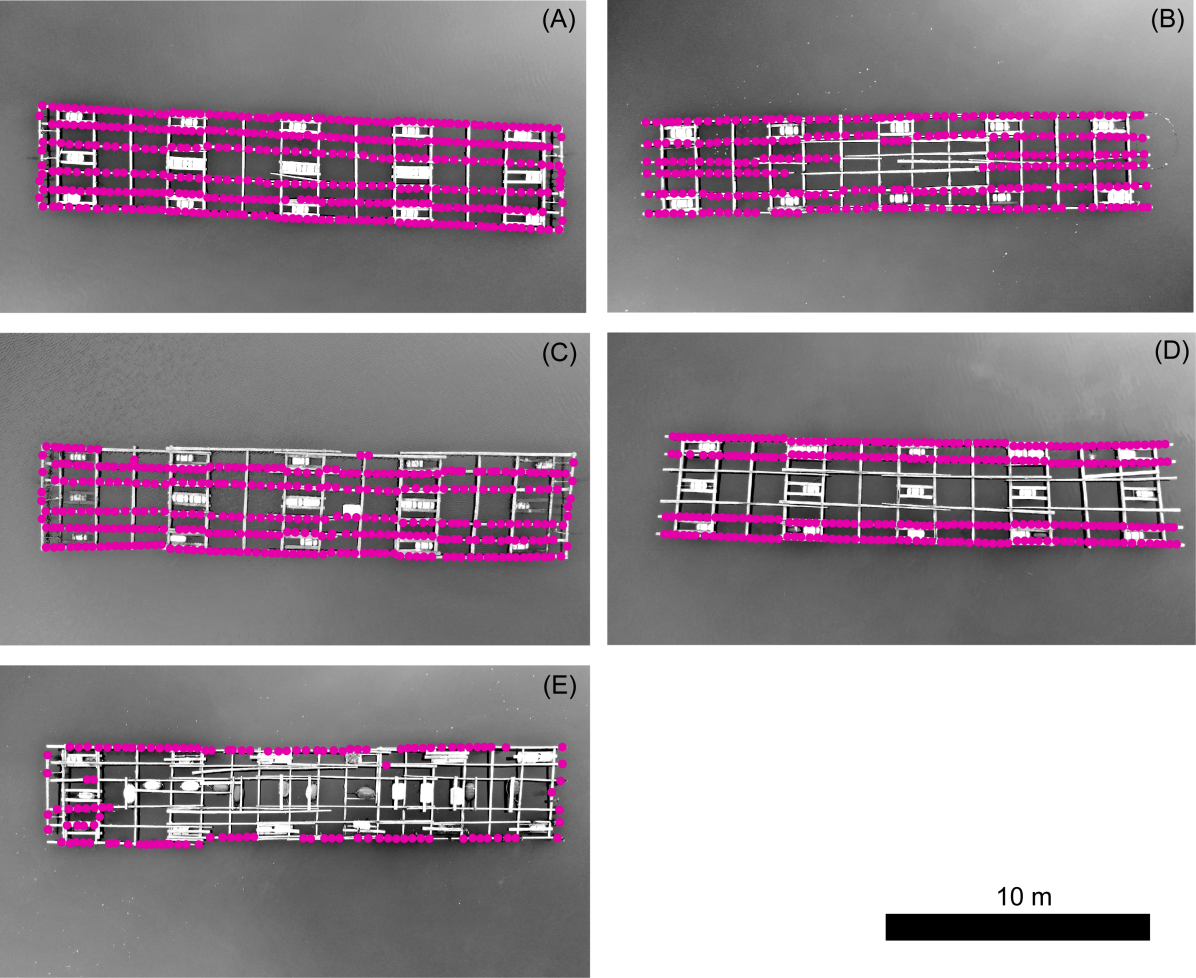
Position of ropes suspending oyster clumps attached to oyster culture rafts in Nagatsura-ura Lagoon. Five photos showing oyster rafts in Nagatsura-ura Lagoon depending on the different number or position of rows to which the rope with the oyster clumps were attached; six rows (A), six rows except center area of the raft (B), five rows (C), four rows (D) and mainly two rows (E). Red dots indicate the positions of ropes suspending oyster clumps. UAV RGB images were converted to monochrome.

The production of shucked oysters from 2007 to 2009 and from 2012 to 2019 were 17,000–31,700 kg and 16,500–41,700 kg, respectively (Fig. 7). Before the tsunami, the production in 2009 was highest during 2007–2009. After the tsunami, the production from 2014 to 2017 was nearly the same or more than in 2009. However, the production decreased in 2018 and 2019 to the same production level in 2008 which was the lowest production during 2007–2009.

### The number of ropes on the oyster culture rafts and their positions on the rafts

Sixty one oyster culture rafts, 63% of the total rafts, were analyzed from aerial images taken by UAV. The number of ropes attached to each raft (Fig. 8) varied from 148 to 498 with an average of 306 (SD = 68). Oyster culture rafts consisted of the different numbers of rows to which ropes with oyster clumps attached and positions of ropes on the raft: six rows (A), six rows except center area of the raft (B), five rows (C), four rows (D) and mainly two rows (E) as shown in Fig. 8 with dots indicating the positions of ropes suspending oyster clumps. The number of ropes attached to six rows was 38, including rafts without ropes attached to the center area of a raft (Figs. 8A–8B). Ropes were attached to either five, four, or two rows in the remaining 23 rafts (Figs. 8C–8E)). Finally, the total raft number of six rows (Fig. 8A) was almost equal to that of others (Figs. 8B–8E).

### Seagrass meadows

**Figure 9:**
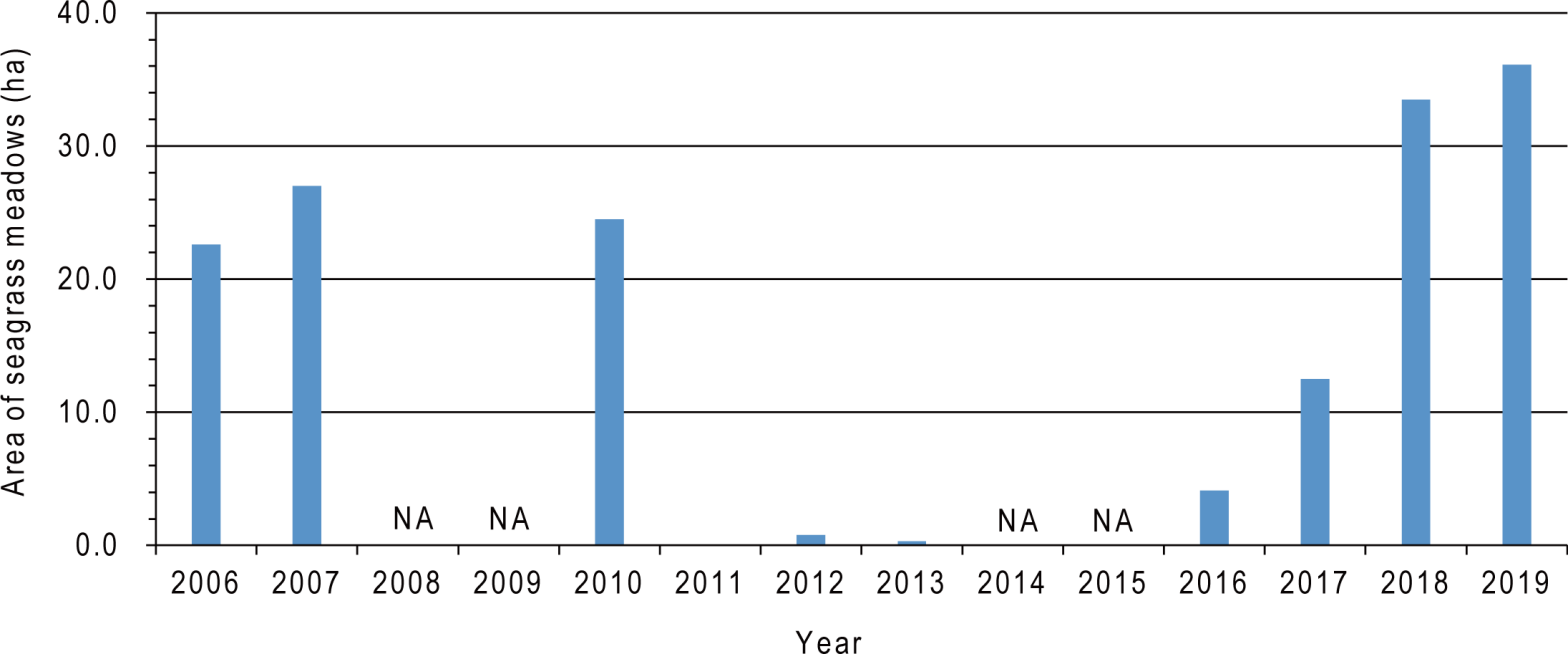
Temporal changes in seagrass meadow area in Nagatsura-ura Lagoon from 2006 to 2019.

We identified the occurrence of the seagrasses *Zostera marina* Linnaeus and *Cladophora* sp. during the field surveys on November 20–22, 2019 (Fig. 2D). The seagrass meadow area before the tsunami from 2006 to 2010 ranged between 22.6 and 27.0, but decreased to < 1.0 ha after the 2011 tsunami and remained at a low level from 2012 to 2013. However, the area gradually increased to 36.1 ha in 2019, which was greater than the seagrass area from 2006 to 2010 (Fig. 9, Table 3). The overall classification accuracy was 71.7% in 2016 and 83.9–85.0% from 2017 to 2019. The kappa coefficient was 0.57 in 2016 and 0.74–0.75 from 2017–2019 (Table 3). Seagrass was not observed on the bottom of the channel along the axis of the bay from south to north (red solid ellipses in Figs. 6H–6J). The seagrass observed in the central-east area (red dotted ellipses in Figs. 6I–6J) in 2017 completely disappeared in 2018 and 2019.

### Coastline changes before and after the tsunami

On April 6, 2011, the seawall, land, and sand beach observed before the tsunami (Figs. 6A–6C) had completely disappeared after the tsunami. Restoration work on land from the tsunami damage began after the 2011 tsunami resulted in the appearance of a temporary cofferdam in the northwest area of the lagoon during 2013 to 2017 (Figs. 6F–6H). A new seawall and breakwater (Figs. 2E–2F) appeared in the northern area on June 7, 2018 and inferring the near-completion of the restoration works on May 8, 2019 (Fig. 6J). The coastline was significantly changed from 2010 (before the tsunami) to 2019 (after the tsunami). Due to the 2011 tsunami restoration work, approximately 6.8 ha of land area converted to water area at the entrance of Nagatsura-ura Lagoon, and approximately 3.0 ha of the water area converted to land area in the inner lagoon. The opening width of the lagoon entrance increased by 4.5 times from ∼40 m before the tsunami to ∼180 m after the tsunami (Figs. 6C and 6J).

## Discussion

On April 6, 2011, after the tsunami on March 11, 2011, 40 (41%) of 98 oyster culture rafts found on June 25, 2010 survived the 2011 tsunami in Nagatsura-ura Lagoon. Some rafts on the sea surface with floating entangled debris were not included in 40 rafts that survived the tsunami and were immediately repaired and for their reuse. Thus, one year later, on February 22, 2012, 63 rafts were observed by addition of 23 new rafts. In Onagawa Bay, the southern part of the Sanriku Coast, all culture longlines for oyster, scallop, and ascidian aquaculture were destroyed by the tsunami (Fujii et al., 2019). The difference in the level of damage between Onagawa Bay and Nagatsura-ura Lagoon may be due to differences in their geographical features. Onagawa Bay is directly exposed to the Pacific Ocean, while a channel connects the Nagatsura-ura Lagoon to Oppa Bay. Nagatsura-ura Lagoon was therefore relatively protected from the direct impacts of the tsunami by the surrounding land and mountains. The number of oyster culture rafts in Nagatsura-ura Lagoon after the tsunami was recovered to that before the tsunami in two years after the tsunami, whereas it took five years after the tsunami for oyster, scallop, and ascidian culture longlines in Onagawa Bay to be restored to 60% of their original level (Fujii et al., 2019). Therefore, the recovery speed and situation along the Sanriku Coast varied depending on the level of damage caused by the tsunami.

Lower chlorophyll *a* content inside than outside the raft indicates that reduction in chlorophyll *a* as a result of oysters’ filter feeding. When ropes are set in wider space, oysters have a chance to filter seawater with more feeds than those on the rope densely set on the raft. Based on the information from oyster culture fishermen, this method was developed by experienced local fishermen to improve oyster growth. Furthermore, the mean area of a raft increased after the tsunami and continued to increase continually from 2012 to 2019. The production of shucked oysters increased from 2014 to 2017. Because the number of rafts from 2013 to 2019 were almost equal to that before the tsunami, it is possible that one of the reasons of high production after the tsunami is the new method developed by the fishermen to space ropes by enlarging the raft size. In Shizugawa Bay north of Nagatsura-ura Lagoon, fishermen decreased the number of oyster culture longlines after the tsunami in 2011 and set longlines at wider space than before the tsunami (Komatsu et al., 2019; Komatsu et al., 2020). However, two demarcated fishing right areas have two different densities of oyster culture rafts (Komatsu et al., 2019; Komatsu et al., 2020). In situ cage experiment of oyster growth indicates that the oyster growth in lower density area is higher than that in higher density area (Sakamaki & Nishimura, 2019). Thus, the method spacing ropes might contribute to increase in oyster production in Nagatsura-ura Lagoon. However, the production decreased in 2018 and 2019 (Fig. 7). This might cause by the construction of breakwaters at the entrance of the lagoon which decreased the water exchange (Figs. 6I–6J). The expansion of seagrass meadows also might cause the decrease in oyster production. Scheffer et al. (2001) suggested that there is a non-linear relationship between nutrient content, phytoplankton content, light supply to the bottom layer, and seagrass/seaweed coverage of the bottom layer. This suggests the expansion of seagrass meadows decreased the nutrient content and subsequently decreased the phytoplankton which are food resources for oyster. It is also conceivable that the expanded seagrass meadows reduced the current velocity (Fonseca et al., 1982) and hindered seawater exchange.

A mass mortality event had occurred once every few years before the tsunami, whereas mortality has not occurred after the tsunami (Hara et al., 2018). Bottom water hypoxia arising from decomposition of organic loads from oysters by bacteria in the bottom surface layer and/or the transport of high turbidity water from the Shin-Kitakamigawa River is thought to be the cause of the mass mortality event before the tsunami (Igarashi, 2006). In Ofunato Bay, the 2011 tsunami destroyed the breakwater at the entrance of the bay and the water quality was significantly ameliorated due to increase of water exchange between the bay and open water (Yamada et al., 2017). Remote sensing on Nagatsura-ura Lagoon reveals the expansion of the lagoon entrance (Fig. 6) after the 2011 tsunami due to land disappearance accompanied with the tsunami and subsidence of the ground level accompanied with the earthquake. Thus, it is possible that the water exchange between the lagoon and Oppa Bay increased by the expansion of the lagoon entrance enables to increase the dissolved oxygen content of seawater after the tsunami, which in turn reduced the occurrence of mass mortality events after the tsunami. A new seawall and breakwater appeared after 2018 might decrease the seawater exchange than before that construction. To prevent mass mortality event, it is essential to maintain high water exchange between Nagatsura-ura Lagoon and Oppa Bay. The breakwater also considered to have the effect to block high turbidity water from Shin-Kitakamigawa River. Thus, civil construction could either degrade or improve the coastal waters and decrease or increase the occurrence of oyster mass mortality event.

Tamaki et al. (2007) conducted field surveys on seagrass meadows in Nagatsura-ura Lagoon and reported a frequent leaking of seagrass and a decline in seagrass meadows from May 2005 to September 2006. They estimated that light intensity at greater depth had decreased due to increased turbidity through organic loads, which then decreased photosynthetic activity of seagrass at greater depths. Simultaneously, the increased sulfide in sediment which cause root rot of plants promoted seagrass leaking. The post-tsunami expansion of seagrass meadows since 2013 suggests that the water quality was improved after the tsunami. It is considered that the expansion of seagrass meadows is due to increase in water transparency by the increased water exchange through the expansion of lagoon entrance similar to the collapse of the breakwater in Ofunato Bay. Therefore, seagrass can grow at deeper bottom depths. Moreover, it may be suggested that the 2011 tsunami likely flushed out deteriorated bottom sediments within the enclosed lagoon in 2011. Despite the increase in total area of seagrass meadows in the lagoon during 2017–2019, seagrass meadow disappeared in area at the central-eastern region of the lagoon where the bottom depth was shallow (red dotted ellipses in Figs. 6I–6J). This might be due to the shallowing of bottom depth, which is caused by ground-level uplift as reported by Geospatial Information Authority of Japan (2020). Thus, continuous monitoring is needed to know seagrass meadow distributions and indirectly health of coastal environment in the lagoon in the future because continuous uplifting and accumulation of organic loads from oyster culture rafts might further increase or decrease the seagrass meadow coverage.

In Akkeshi Lake, Hokkaido, Smith et al. (2018) found that the cultured oysters fed on epiphytic diatoms and epiphyte propagules before they settled on seagrass, which reduced epiphyte loads and influenced subsequent faunal settlements. As a result, the presence of cultured oysters might indirectly affect seagrass by changing the composition and relative abundance of species such as sessile polychaetes, gastropods, and amphipods. Thus, the spatial distribution of oyster culture rafts is beneficial to the seagrass meadows in Nagatsura-ura Lagoon because these were not overlapped. Additionally, the post-tsunami expansion of seagrass meadows in the lagoon could increase the production of oyster because seagrass meadows provide substrates to epiphytic diatoms which are food resources for oyster in lagoon. The expansion of seagrass meadows might support coastal aquaculture not only for food supply but also another positive effect on aquaculture, for example, oxygen production and nutrient absorption. A recent study found that the bloom of the fish-killing raphidophyte flagellate *Chattonella antiauq* had suppressed by a *Chattonella* growth-limiting bacteria living on seagrass biofilms (Inaba et al., 2019). Onishi et al. (2014) found that the seagrass *Zostera marina* harbors growth-inhibiting bacteria against the toxic dinoflagellate *Alexandrium tamarense*. The increased seawater exchange due to the changes in coastline morphology of Nagatsura-ura Lagoon seems improved the coastal environments including filter feeders of coastal aquaculture and organisms such as *Zostera marina*.

## Conclusions

Our study reveals that the key for realizing the healthy and sustainable lagoon environment of oyster culture is to maintain high water exchange between Nagatsura-ura Lagoon and Oppa Bay. Since the local governments’ decisions regarding civil work designs could either degrade or improve the condition of coastal waters, thematic maps showing spatial–temporal changes in aquaculture facilities and seagrass meadows including coastline can contribute to the decisions in ria-type bays. Furthermore, they can support not only decision makers but also stake holders such as fishermen, local cooperative and commune by providing clear visualization for understanding of their temporal changes (Fig. 6). In conclusion, our study in Nagatsura-ura Lagoon show that remote sensing is a practical and essential tool for the sustainable development of coastal waters by contributing to conserve coastal ecosystems and manage aquaculture.

## Additional Information and Declarations

### Field Study Permissions

The following information was supplied relating to field study approvals (i.e., approving body and any reference numbers): We had a verbal agreement from Mr. Kazushi Abe, who is a manager of Kahokucho Branch of the Miyagi Fishery Cooperative Association, to research the oyster culture rafts.

### Data Availability

The following information was supplied regarding data availability: Oyster culture rafts images from UAV are available at Figshare: Murata, Hiroki (2020): UAV.zip. figshare. Figure. https://doi.org/10.6084/m9.figshare.12649166.v1. Maxar Technologies images can found at:
https://discover.digitalglobe.com/
Image ID: 10100100054EA000 (November 9 2006): https://api.discover.digitalglobe.com/show?id=10100100054EA000
Image ID: 10100100059D3300 (May 8 2007): https://api.discover.digitalglobe.com/show?id=10100100059D3300
Image ID: 1050410001B22900 (June 25 2010): https://api.discover.digitalglobe.com/show?id=1050410001B22900
Image ID: 1050410000115D00 (February 22 2012): https://api.discover.digitalglobe.com/show?id=1050410000115D00
Image ID: 105041000299A400 (April 14 2013): https://api.discover.digitalglobe.com/show?id=105041000299A400
Image ID: 104001001B459F00 (April 19 2016): https://api.discover.digitalglobe.com/show?id=104001001B459F00
Image ID: 1030010067471500 (March 19 2017): https://api.discover.digitalglobe.com/show?id=1030010067471500
Image ID: 104001003D54C200 (June 7 2018): https://api.discover.digitalglobe.com/show?id=104001003D54C200
Image ID: 1030010090BDA400 (May 8 2019): https://api.discover.digitalglobe.com/show?id=1030010090BDA400.
